# The influence of vergence facility on binocular eye movements during reading

**DOI:** 10.16910/jemr.12.4.9

**Published:** 2019-12-16

**Authors:** Remo Poffa, Roland Joos

**Affiliations:** 1 University of Applied Sciences and Arts Northwestern Switzerland School of Engineering, Institute of optometry

**Keywords:** eye movement, eye tracking, fixation disparity, associated phoria, dissociated phoria, vergence facility, accommodative facility, reading

## Abstract

Optometrists regularly use binocular measurements in patients with asthenopic complaints when performing close-up work. The focus of this work was therefore on the correlation of optometric parameters and objective fixation disparity (FD) measured by an eye tracker.

In our investigation, 20 participants (6 male, 14 female) were subjected to a classical optometric procedure. Subsequently, these subjects read various sentences on a screen and eye movements were registered by using a RED500 eye tracker. The experiment was performed under two reading distance conditions. In order to be comparable with previous work, the present study was conducted under dark illumination conditions [12]. FD values were deduced from objective eye tracking data during reading. Data analysis was done using linear mixed-effects models.

FD was found to depend on vergence facility (t=3.3, p=0.004). Subjects with a low vergence facility showed more eso fixation disparity than subjects with a normal vergence facility.

If studies of binocular coordination using eye tracking methods are performed under dark illumination conditions, vergence facility is an important parameter and should be accounted for. Neglecting this parameter may mask other important parameters. Vergence facility in context of reading difficulties may be important.

## Introduction

In the last few years, investigating binocular eye movements has become increasingly more interesting. Due to binocular eye trackers and better resolution, interest in binocular coordination during reading has raised. Studies have been done in topics like reading processing [1-3], binocular coordination [2, 4-10], and many others. In these studies, subjects were measured on different eye trackers, primarily binocularly in different reading distances and under different background illuminations.

Previous studies have reported conflicting results recording fixation disparity [2, 11]. Kirkby investigated whether the type of eye-tracker can be a reason for different results [12]. This study showed comparable results for both eye-trackers in similar experimental settings. Under dark luminance conditions, Kirkby found a majority of uncrossed fixations. Due to the fact, that dual Purkinje eye tracker (DPI) measurements can be done under dark room conditions only, her study is restricted to dark background illumination.

The before mentioned studies are important for understanding coordination of eye movements in reading and for understanding how visual information in reading is processed.

In all studies previously cited, subjects had normal vision or had been corrected to normal far vision prior to starting the experiments. Mostly, optometrists or ophthalmologists not involved in the study had done the eye exams. Moreover, the exams were completed weeks or months before the study was performed and if screening was included at the beginning of the study, this screening was comprised of only visual acuity (VA) and stereo vision (SV).

Hung developed a model of the interaction between accommodation and vergence [13]. The influence of this interaction has been investigated in different studies. [14-16]. These studies show that when accommodation occurs, vergence more or less automatically happens. From a purely physical point of view, the relation between accommodation and vergence should be unique. Because of physiological variations, this is not the case. The relation between accommodation and vergence is measured by the AC/A ratio [17].

Hyperopic patients can still have a normal VA and SV by compensating their hyperopia with accommodation. Because of the coupling of accommodation and vergence, this induces a convergence movement of the eyes and therefore a crossed visual axes condition. For various reasons it is possible that uncrossed visual axes may occur.

The state when the visual axes of both eyes do not intersect at the focus plane, still producing a single image while crossing before (esophoria) or behind (exophoria), is called fixation disparity [18].

It must be assumed that the interactions between refractive state, accommodative state and vergence state influence binocular coordination of fixations and saccades. To be able to assess the effect of this interaction of binocular coordination, it is essential to check each participant regarding his accommodative and vergence system.

Alvarez and Kim [19] analyzed peak velocity to symmetrical convergence stimuli. Subjects with convergence insufficiency (CI) and controls with normal binocular vision showed different vergence behavior for symmetrical convergence step stimuli between 2° and 12° in 2° steps. They could verify that subjects with CI exhibited differences in the peak velocity between the left eye and right eye, whereas subjects of the control group did not show such differences.

It cannot be excluded that refractive state and binocular state, as well as other optometric variables, may have influence on eye-tracking results. Therefore, a thorough optometric testing of each test participant was performed in the test setup applied in this study.

The goal of this study was to investigate if implementing optometric variable “vergence facility” influences eye fixations under dark illumination conditions. Vergence facility is an easy optometric test to measure convergence and divergence responses. A second goal was to show how experimental conditions influence FD results. For this reason, an experimental setting was considered that allowed for different reading distances.

## Methods

Prior to the experiment, subjects were carefully checked on their refractive, accommodative and vergence states by optometrists. The following screening tests have been included in the battery:

Habitual correction (HC), visual acuity (VA) far with HC, VA near with HC, cover test far, cover test near, phoria 6m, associated phoria 6m, dissociated phoria 40cm, AC/A ratio, vergence facility, accommodative facility.

Visual acuity was measured on a DMD Pola Vista Vision at 6 meters for far distance and with the Zeiss Polatest for near distance at 40cm. Visual acuity (VA) far and near with habitual correction (HC) was at minimum 20/25 in all subjects.

The cover test is a classical optometric test to see objectively a tropia. The uncover test in turn shows a phoria. It can be done in far and near distance [17].

Phoria for distance (6m) was measured in prism diopters (pdpt) on a polarized test from the MCH (Measuring and Correcting Methodology after H.-J. Haase) test battery showing a cross [20].

Associated phoria for distance (6m) is defined as the amount of prism required to reduce subjective fixation disparity to zero [17]. For this measurement we again used a test from the MCH test battery (cross test, pointer test) [20].

For near distance (40cm) we measured the dissociated phoria with Modified Thorington Test [17].

AC/A ratio is a measure of the interaction between accommodation and vergence. It indicates the amount of convergence a subject needs when he has to accommodate a certain amount (normally 1D). A normal AC/A ratio is 4:1 (+/-2) [17].

Vergence facility (VF) is a test to measure the flexibility of the vergence system [17, 21].

Therefore, a combination of two prisms with the values 12 pdpt base out and 3 pdpt base in were used (Bernell vergence facility prism, Item#: G1110+). Subjects were asked to fixate a vertical column of 20/25 letters in 40cm and asked to try to keep the column single and clear. Prism (3δ (base in (BI)) was introduced first and the subject asked to report when it became single. When the target was single and clear, the 12δ (base out) BO was introduced. When the subject reported that the target was clear, the prism was switched back to the 3δBI. This was repeated for one minute and the number of cycles was noted. Normal range of vergence facility in 40cm is 12-18 cycles per minute (cpm) [17], but a larger facility is not conspicuous.

The flexibility of the accommodation was done by accommodative facility (AF) testing [17]. The subject fixated a vertical column of 20/25 letters in 40cm and had them to keep clear and single. Accommodative facility was assessed in cpm, both monucularly and binocularly, using flipper lenses +2.00/-2.00 (Bernell accommodative flipper, Item#: BC1270+). This was repeated for one minute and the number of cycles was noted. Normal range of accommodative facility in 40cm is 10cpm (+/-5) [17], but again a larger facility is not conspicuous.

After the optometric screening, it was stated that all subjects had binocular vision and no strabism. We could not exclude convergence insufficiency (CI), because neither near point of convergence nor positive and negative fusional vergence ranges were measured. None of the subjects mentioned learning disabilities.

The experiment was set up where reading distance was varied as follows: Subjects had to proceed a reading task on a screen from a distance of 100cm and 50cm under dark illumination condition binocularly. For clarification, white text was presented on a screen with a black background, with no ambient light sources. Text consisted of 40 sentences from the Potsdam Sentence Corpus [2]. There were two trials, one for each distance; within each trial, subjects had to read 4 blocks consisting of 5 sentences. To keep the subjects concentrated on their task, they answered an easy question after each block by knocking on the table to signalize yes or no.

Eye movements have been tracked binocularly during this reading task.

## Subjects

29 students expressed their interest to take part in the study. An optometrist carefully checked each student. Nine students were excluded after the measurements because of technical problems using the eye tracker with high index glasses.

20 adult subjects were enrolled in the experiment, 14 females and 6 males. All were students at the University of Applied Sciences Northwestern Switzerland at the Institute for Optometry. Seven wore glasses, 2 of whom were male. 16 had German as their first language and 4 French, 2 of them were male. All subjects fluently read German. They were paid CHF 40 in cash for volunteering.

In order to obtain results of good quality, manufacturer’s validation sequence had to be within +/-1°. Due to problems during this sequence (small head movements, inaccurate fixation and narrow lid opening), data from 18 subjects at 50 cm working distance could be analyzed. For the experiment at 100cm working distance, only data from 8 subjects remained for analysis.

## Materials

We used the RED 500 remote eye tracking system from SMI for recording binocular eye movements. The manufacturer’s software I View X version 2.8 was installed on a Dell Latitude E6530 Laptop. All experiment stimuli were presented on a Packard Bell Full HD 21.5-inch screen with 1920x1080 px resolution. Text blocks, consisting of 5 sentences, were presented in Courier New font size 25 for 100cm (1 character space 0.27°) and 14 for 50cm (1 character space 0.27°) screen distance. All sentences were chosen from the Potsdam Sentence Corpus [2]. One screen of text contained 5 lines; for each line of text an individual number of fixations was recorded. To identify fixations per line they were attributed a “fixation order sequence number”. To keep distance constant, a chin- and forehead rest was used. White text was presented on a black background, in a dark room with no ambient light sources. Luminance was measured with a Minolta luminance meter LS-110. Luminance did not change during the entire experiment. The luminance conditions were similar to Liversedge et al. [22].

The manufacturer declares spatial resolution of its eye tracker with 0.03°, i.e. 1.8 arcmin. The Red500 eye tracker can be operated at 500, 250,120 and 60Hz. Pilot measurements resulted in data with lower noise when it operated on 250Hz (internal study, not published).

Calibration was done binocularly at the start of every trial. First, the subjects performed the manufacturer’s calibration and validation sequence. If the manufacturer’s validation was within a tolerance limit of +/-1°, a specific calibration sequence was done. This second calibration procedure was done in order to account for the fact that only the central part of the screen was used and to refine the manufacturer’s calibration. A particular feature of this second calibration was the possibility to determine phoria for each eye separately using dissociated stimuli, i.e. stimuli that could be seen by either the right or the left eye only.

The experiments were programmed with PsychoPy V 1.80.04, a python-based programming environment. The sequence order of the 4 experiments was balanced on the 20 subjects. Eye movement data from all subjects were collected in the same laboratory within the Institute of Optometry at the University for Applied Sciences Northwestern Switzerland. Testing sessions took between 30 and 45 minutes for each participant, depending on the time needed to get sufficient calibration accuracy. Prior to the experiment, subjects were instructed to read each sentence normally for comprehension. They indicated by fingertip when they reached the end of each block. Each reading block was followed by a question, which tested the comprehension of the text. After answering the question by knocking on the table, the next block to read appeared. After 4 blocks, a short relaxing break was taken before the next experiment started.

## Design

In-house software programmed in R [23] was used to analyze the data. Raw horizontal position output from the RED 500 was converted from pixels to degrees. Streams of raw data were used and fixations and saccades were identified manually. Fixation disparity (FD) was calculated by subtracting the horizontal position of the right eye from that of the left eye at the start, the center, and the end of each fixation. The FD was calculated as the mean of these three values. Crossed fixations were those where the left eye’s point of fixation was more to the right of the right eye’s point of fixation and uncrossed fixations where the left eye’s point of fixation was more to the left of the right eye’s point of fixation. Fixations less than 80ms or more than 1200ms and FDs of a magnitude of more than 1.5° were considered as invalid or outliers, and therefore were removed from the analysis. After this filtering, 81.15% of 9077 fixations remained and subsequently underwent further analysis. The number of fixations needed to read an individual line of text varied considerably between subjects and reading conditions. In order to avoid overestimation of the effects of high fixation sequence numbers, analysis was restricted to 8 fixations per line.

Statistical analysis was done with R [23] using package “nlme” [24]. Linear mixed models are appropriate to deal with these kind of repeated measures data. Dependent variables typically were FD and independent variables, i.e. factors and covariates, were working distance and vergence facility, while test subjects were treated like “random factors”. Non-significant terms in linear mixed models were eliminated according the principle of parsimony and appropriate significance tests. Results are presented with relevant regression t-tables.

## Results

All 29 subjects underwent an optometric screening.

From all 29 subjects undergoing the optometric tests, 9 had to be excluded as previously explained.

From the remaining 20 subjects, 11 subjects did not need glasses. All of them were moderate myopes and none had any substantial anisometropia (<0.5D). Three subjects were wearing prismatic corrections. One of them 6 prism diopters (pdpt) base in (BI), one 1 pdpt BI and one 7 pdpt base out (BO).

Tables 1 to 5 show the distribution of the 20 subjects divided according to their habitual correction or the direction of their phoria.

**Table 1:** Range of habitual corrections in diopters (dpt)

	sphere	cylinder
OD	-0.25 - -2.75	-0.25 - -0.75
OS	-0.75 - -2.25	-0.25 - -0.75

**Table 2:** Distribution of far and near cover test.

	subjects
eso far/near	4
ortho far/eso near	2
exo far/eso near	1
exo far/near	9
ortho far/exo near	4

**Table 3:** Distribution of distance phoria (6m)

	subjects	range in pdpt
orthophoria	7	-
exophoria	7	1 - 5
esophoria	6	1 - 9

**Table 4:** Distribution of associated phoria (6m)

	subjects	range in pdpt
exophoria	5	0.5 – 4.5
esophoria	4	1 - 11

**Table 5:** Distribution of dissociated phoria (40cm)

	Subjects	range in pdpt
orthophoria	4	-
exophoria	10	0.5 - 6
esophoria	6	1 - 9

The tables 6 to 8 show the distribution subdivided according to their dissociated phoria (40cm).

**Table 6:** Distribution of AC/A ratio

subjects	low AC/A	normal AC/A	high
orthophoric	2	2	0
exophoria	3	7	0
esophoric	0	5	1

**Table 7:** Distribution of vergence facility

subjects	low VF	normal VF	high VF
orthophoric	1	3	0
exophoric	7	1	2
esophoric	4	2	0

**Table 8:** Distribution of accommodative facility

subjects	low AF	normal AF	high AF
orthophoric	1	3	0
exophoric	6	4	0
esophoric	4	2	0

Fixation disparity (FD) was regarded as a dependent variable; a variety of factors and covariates like gender, age, refractive state, AC/A etc., i.e. essentially all optometric variables described before, initially were included in the linear mixed-effects model. Removing all factors not contributing to the goodness of fit or not being significant, finally led to a model with one significant covariate only, i.e. vergence facility.

Surprisingly, reading distance is not significant in this linear mixed-effects model and therefore is not contained in table 9.

**Table 9:** Regression table for the linear mixed-effects model for the FD.

	value	SE	DF	t	p
(Intercept)	0.28	0.086	1907	3.27	0.001
VergFlex	-0.03	0.009	15	-3.37	0.004

**Figure 1:**
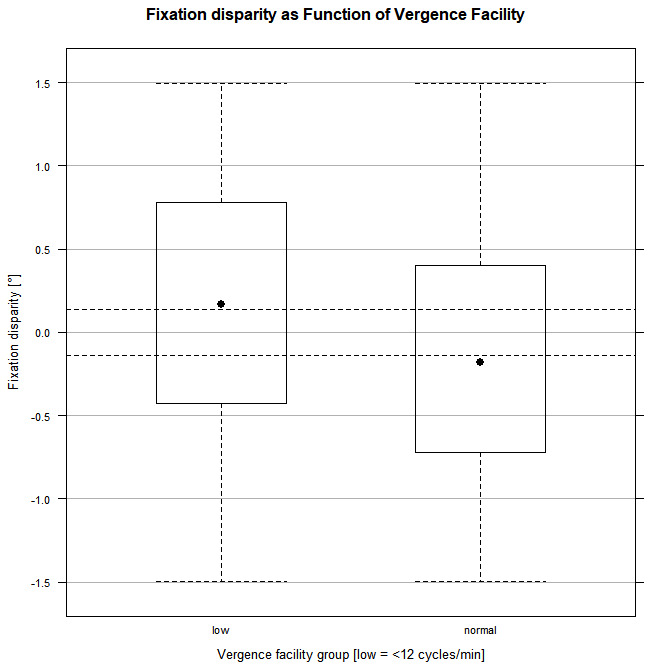
dashed lines indicate +/- ½ character space (+/- 0.14°) distance between intersection points of fixation lines in the target plane.

In order to visualize the results, two vergence facility groups were constructed, according to optometric practice, where vergence facility values below 12 cpm are considered as “low” and values equal to or greater than 12 are classified as “normal”. Figure 1 shows a mean FD for the low vergence facility group (n=12, mean= 1.5 cpm) of 0.145° esophoria (sd=0.764) and for the normal group (n=8; mean= 15 cpm) of 0.139° exophoria (sd=0.740). The difference corresponds roughly to one-character space, as can be seen in figure 1.

The regression table (Table 9) shows an intercept value of 0.28. This means that a participant with zero cyles per minute (cpm) of vergence facility would have a FD of 0.28° esophoria (t=3.27, p=0.001). The vergence facility “VergFlex“ value of -0.03 (t=-3.37, p=0.004) shows the change in FD in degree per cpm. Since this value is negative, the change goes in the direction of exophoria. Model evaluation for the “normal” vergence facility group would result to -0.42° of fixation disparity.

It may be surprising that the working distance was not a statistically significant covariable; this may be because only 8 test subjects gave reliable results for the 100cm test distance. Therefore, this finding deserves no emphasis.

It can be summarized that the linear mixed-effect model shows a clear and statistically significant effect of the optometric variable vergence facility.

## Discussion

Kirkby’s results suggest that experiment conditions, such as luminance and viewing distance, influenced variability in previous studies of binocular fixation alignment [12]. The aim of this study was to investigate whether optometric variables like “vergence facility” influence fixation alignment under dark illumination conditions and to show in which way this parameter is attributable to FD results.

Figure 1 reveals that for normal vergence facility, mean FD was more uncrossed. For this group we have good agreement with Kirkby [12]. On the other hand, lower vergence facility is correlated with more crossed fixation. This seems to be in conflict with the finding of more exophoria in the work of Kirkby.

It could be hypothesized that low vergence facility cases tend to stay closer to the rest position of vergence under dark illumination conditions, that is generally more eso, than normal vergence facility cases [25] .

It is well known that accommodative state and vergence state are correlated. This is because accommodation and vergence must be well coordinated in order to get clear binocular vision at different viewing distances. According to Leibowitz [26], the rest position of accommodation under dark illumination conditions tends to be at finite distance.

Therefor any participant with low vergence facility would tend to assume a closer rest position of accommodation and vergence before starting reading. It then tends to compensate the misalignment of the visual axes in order to improve binocular perception. On the contrary, subjects with normal vergence facility start with a more distant accommodative focus and vergence alignment and must compensate for this when reading a single line of text.

One possible explanation of the finding of the present study could be that the state of vergence is only partially adjusted after starting the reading task; hence, the low vergence facility subjects would tend to remain rather esophoric, while normal vergence facility subjects would tend to remain more exophoric.

Alvarez and Kim [19] investigated convergence velocity in subjects with convergence insufficiency (CI). They found a significant asymmetry of the peak velocity to symmetrical convergence stimuli compared to a binocularly normal control group. This may show that the dynamic behavior of convergence is related to convergence problems. Therefore, it is not surprising that vergence facility, obviously a dynamic property of vergence, does also affect FD.

Although this thought seems to be straightforward, there are limitations. First, it should be stated that Alvarez and Kim worked with bright light conditions. Second, in our study we did not measure CI. So, no direct link between CI, VF and FD can be deduced from our work.

While Alvarez and Kim [19] focused their work on CI, others investigated vergence facility in subjects with reading difficulties or reading problems caused by dyslexia. Dusek investigated schoolchildren with reading difficulties in Austria. He found a significantly lower vergence facility rate in children affected by reading difficulties compared to a control group without reading difficulties [27]. Buzzellli found in dyslexic subjects a significantly lower vergence facility rate than in the normal control group [28]. To summarize, there are many indications in literature that vergence facility rate correlates with reading difficulties.

On the other hand, reading difficulties also correlate with fixation disparity or dyslexia [29, 30]. Therefore, low vergence facility could be the common cause of reading difficulties and fixation disparity.

In an experimental setup that tries to control all factors that may influence fixation disparity, one would have to measure and record the vergence state under dark and bright illumination conditions. This would afford a considerable amount of time that may result in unwanted fatigue of the subjects. Instead of doing so, one could use vergence facility that could serve as an easy to use and time sparing screening variable.

## Ethics and Conflict of Interest

The investigation adhered to the principles of the Declaration of Helsinki. All subjects had to sign a written declaration of agreement to participate in a clinical study (declaration form: study group of Swiss research- and ethic commissions for clinical studies).
